# Supplementary figures and images for: Cross-sectional association between blood cholesterol and calcium levels in genetically diverse strains of mice
Source: bioRxiv. 2023 Feb 9:2023.02.08.527123. Preprint. [Version 1] doi: 10.1101/2023.02.08.527123 (PMC9934644; doi:10.1101/2023.02.08.527123)

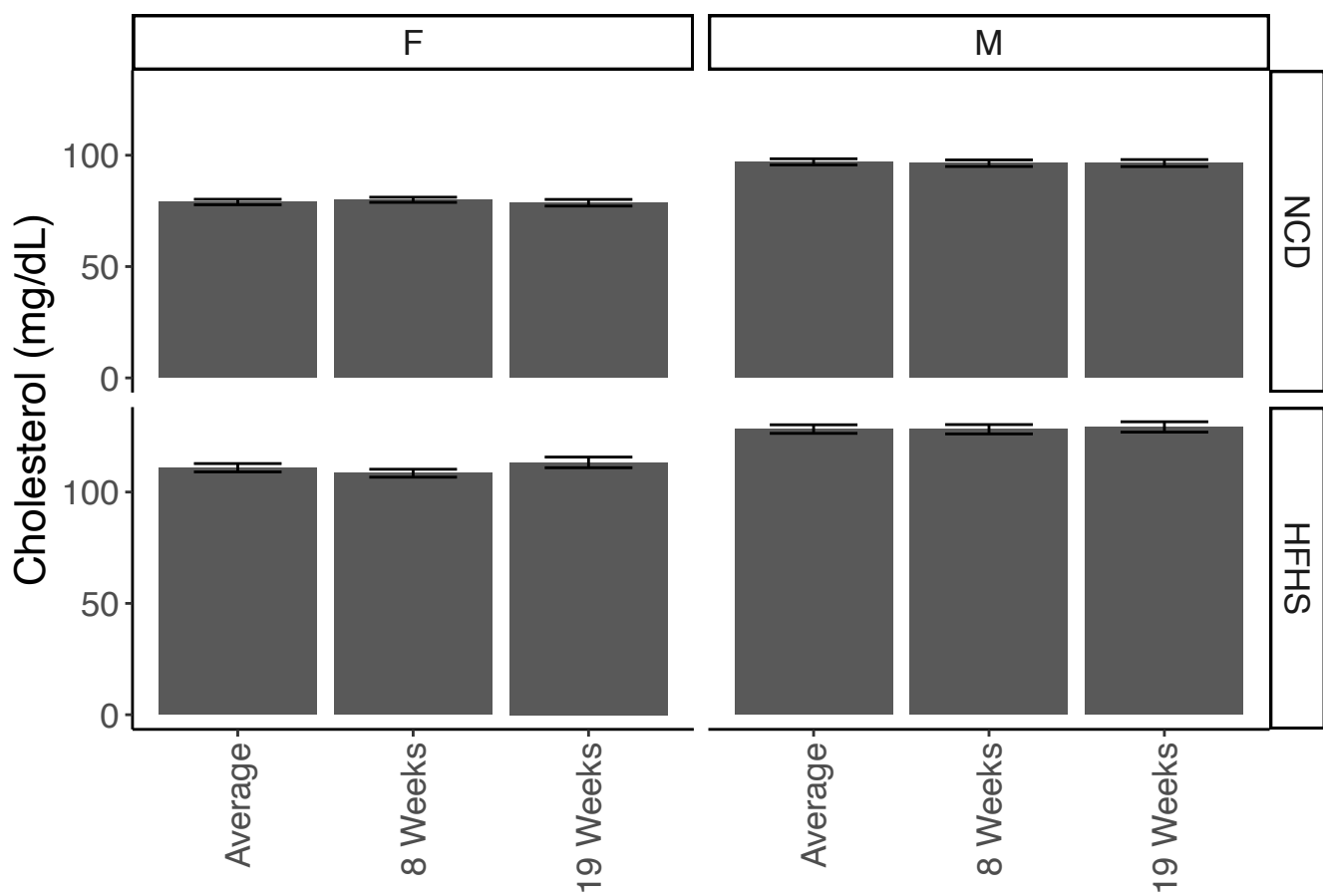

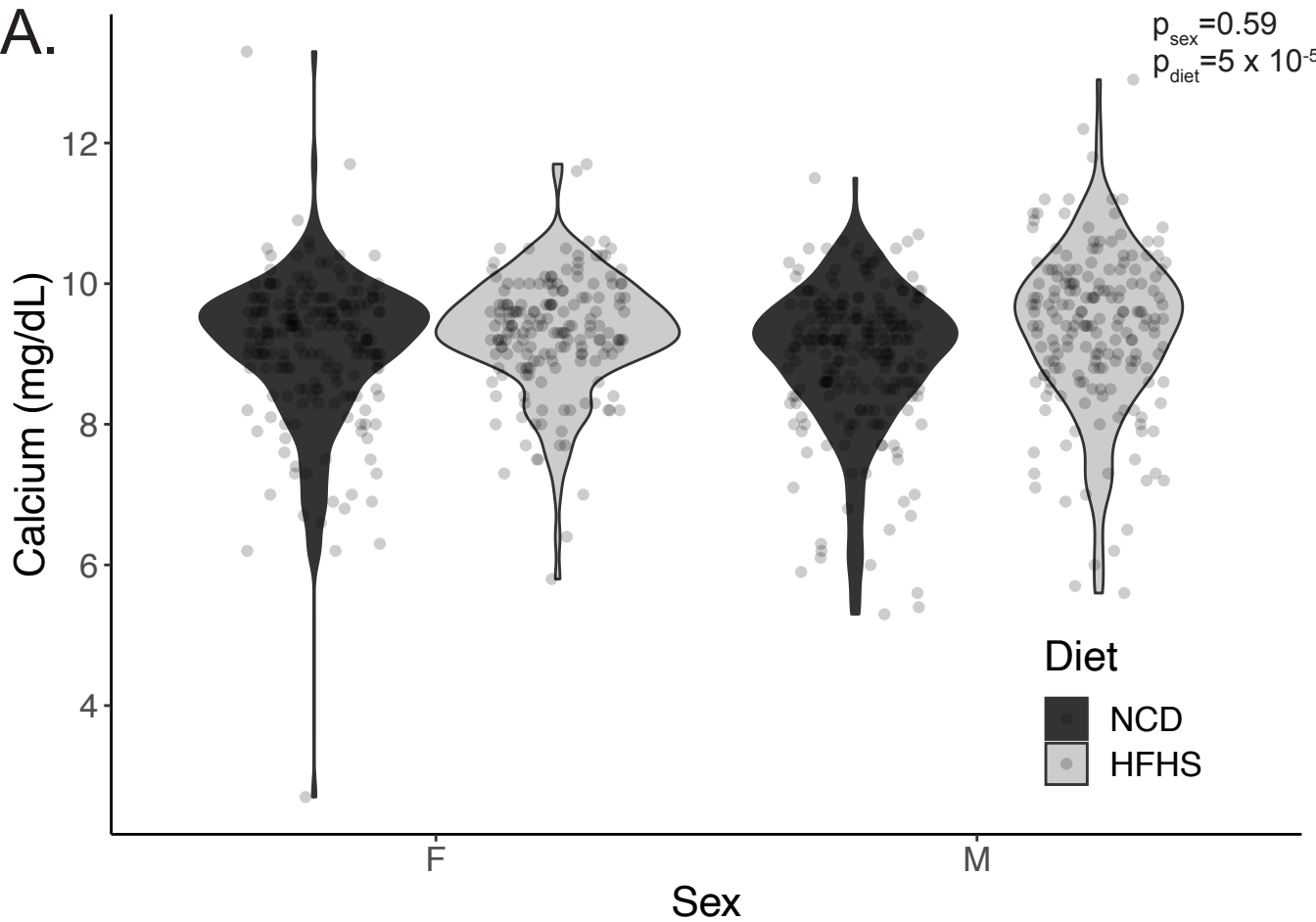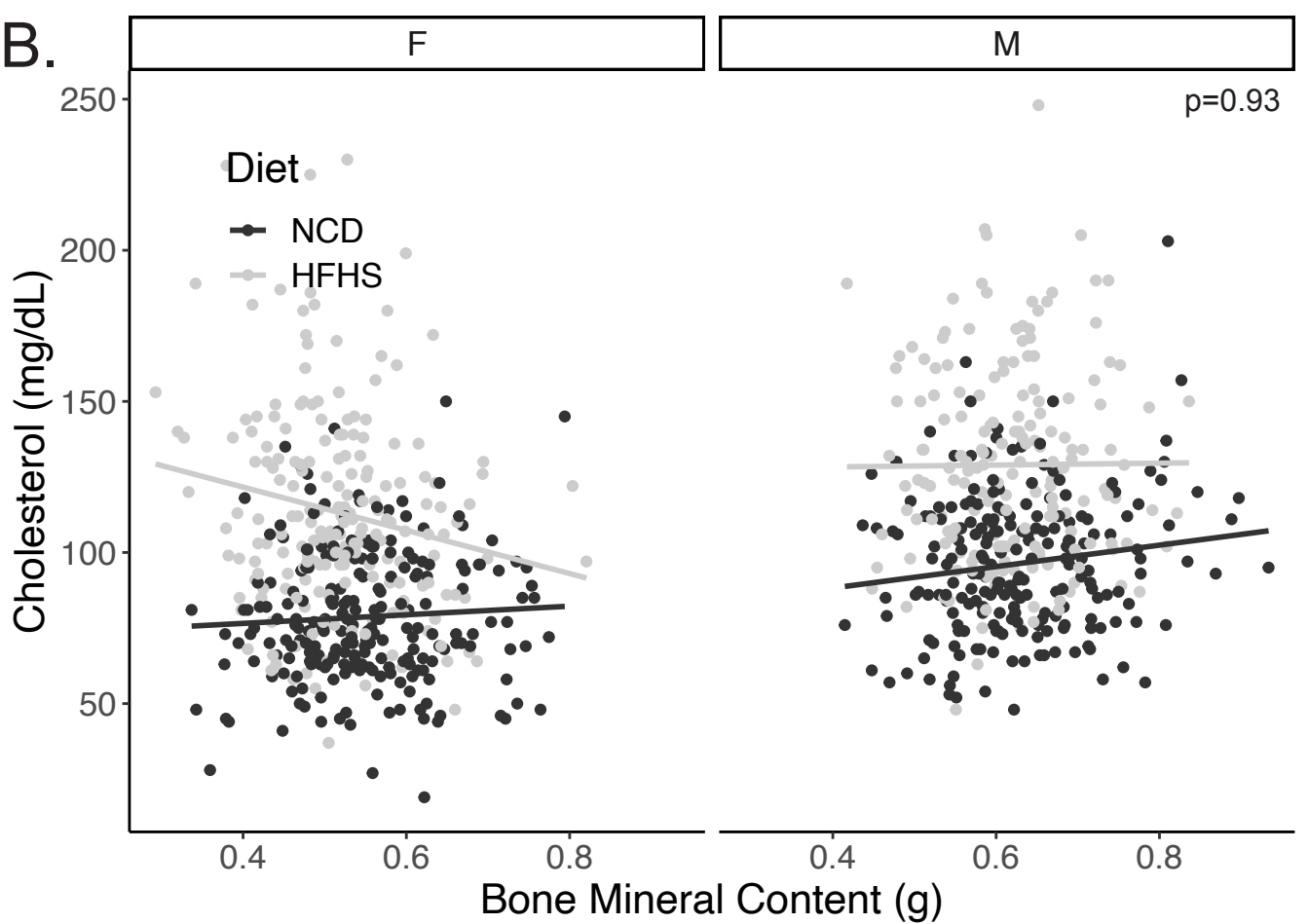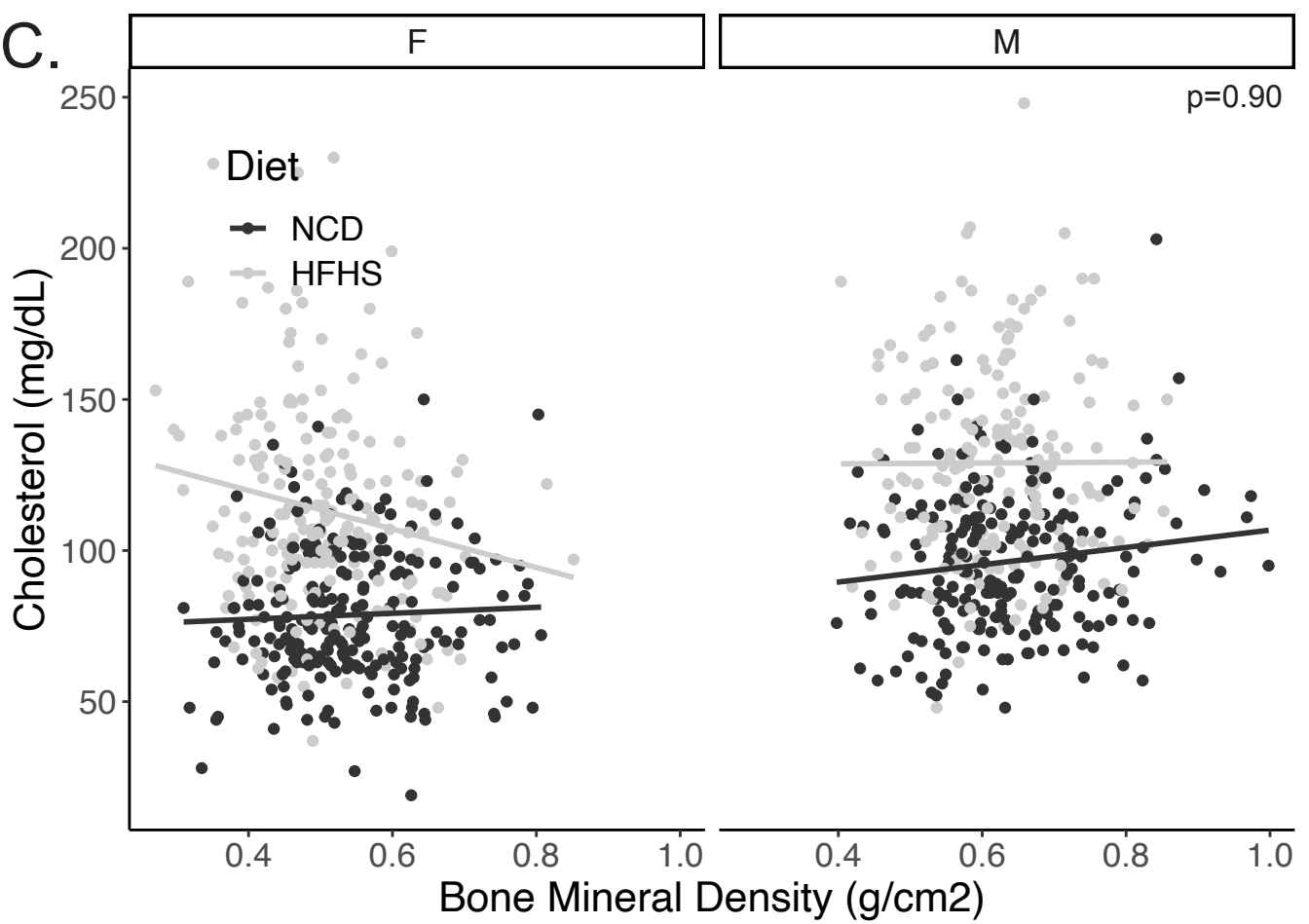

Supplement: Supplement 2 [file media-2.pdf]
